# Osmoregulation in Barnacles: An Evolutionary Perspective of Potential Mechanisms and Future Research Directions

**DOI:** 10.3389/fphys.2019.00877

**Published:** 2019-08-21

**Authors:** Kristina Sundell, Anna-Lisa Wrange, Per R. Jonsson, Anders Blomberg

**Affiliations:** ^1^Department of Biological and Environmental Sciences and Swedish Mariculture Research Center (SWEMARC), University of Gothenburg, Gothenburg, Sweden; ^2^IVL Swedish Environmental Research Institute, Fiskebäckskil, Sweden; ^3^Department of Marine Sciences, Tjärnö Marine Laboratory, University of Gothenburg, Gothenburg, Sweden; ^4^Department of Chemistry and Molecular Biology, University of Gothenburg, Gothenburg, Sweden

**Keywords:** osmoregulation, molecular mechanisms, euryhalinity, Na^+^/K^+^-ATPase, aquaporins, Crustacea, Cirripedia

## Abstract

Barnacles form a globally ubiquitous group of sessile crustaceans that are particularly common in the coastal intertidal. Several barnacle species are described as highly euryhaline and a few species even have the ability to colonize estuarine and brackish habitats below 5 PSU. However, the physiological and/or morphological adaptations that allow barnacles to live at low salinities are poorly understood and current knowledge is largely based on classical eco-physiological studies offering limited insight into the molecular mechanisms. This review provides an overview of available knowledge of salinity tolerance in barnacles and what is currently known about their osmoregulatory strategies. To stimulate future studies on barnacle euryhalinity, we briefly review and compare barnacles to other marine invertebrates with known mechanisms of osmoregulation with focus on crustaceans. Different mechanisms are described based on the current understanding of molecular biology and integrative physiology of osmoregulation. We focus on ion and water transport across epithelial cell layers, including transport mechanisms across cell membranes and paracellular transfer across tight junctions as well as on the use of intra- and extracellular osmolytes. Based on this current knowledge, we discuss the osmoregulatory mechanisms possibly present in barnacles. We further discuss evolutionary consequences of barnacle osmoregulation including invasion-success in new habitats and life-history evolution. Tolerance to low salinities may play a crucial role in determining future distributions of barnacles since forthcoming climate-change scenarios predict decreased salinity in shallow coastal areas. Finally, we outline future research directions to identify osmoregulatory tissues, characterize physiological and molecular mechanisms, and explore ecological and evolutionary implications of osmoregulation in barnacles.

## Salinity and Barnacles

Salinity plays an important role in shaping aquatic communities (Gunter, 1961; Remane and Schlieper, 1971; Whitfield et al., 2012). Barnacles (Cirripedia, Crustacea) are like many other crustaceans among the few marine invertebrates that can invade brackish habitats (Davenport, 1976), with species like *Balanus (Amphibalanus) improvisus* and *Balanus (Amphibalanus) subalbidus* tolerating almost freshwater conditions (Fyhn, 1976; Poirrier and Partridge, 1979; Kennedy and di Cosimo, 1983; Dineen and Hines, 1994). Already Darwin (1854) noted that several barnacle species were euryhaline, i.e., displaying a high tolerance to a wide range of salinities. Many barnacle species are also common fouling organisms, e.g., on ship hulls (Otani et al., 2007), where broad salinity tolerance has been a key trait for surviving long-distance transportation, making them successful invaders worldwide and causing major ecological and economic impact in coastal areas (Lewis and Coutts, 2009).

Estuarine environments are characterized by temporary, strong environmental fluctuations in salinity, which greatly affect the organisms living there. Few species are adapted to a life in estuarine environments, which is reflected in low biodiversity compared to marine or freshwater habitats (Remane and Schlieper, 1971). In addition, many estuaries, brackish fjords and regional seas, e.g., Chesapeake Bay and the Baltic Sea, constitute ecosystems that are rapidly deteriorating through habitat destruction, pollution, and climate change (Diaz and Rosenberg, 2008; Halpern et al., 2008). Loss of biodiversity in these already depauperate, brackish ecosystems threatens the provision of many ecosystem services that are necessary for human welfare and economic development (Worm et al., 2006). Many coastal areas are also expected to become less saline due to increased precipitation and freshwater run-off, driven by global warming (MacKenzie et al., 2007; Najjar et al., 2010). Management, conservation, and potential restoration of coastal areas under current and future environmental changes call for an improved understanding of how biodiversity is affected by the organism’s tolerance to brackish water conditions. Barnacles display a wide range of abilities to cope with environmental changes including salinity, and therefore provide an interesting framework to study the evolution of osmoregulatory functions as well as their ecological importance for ecosystem functioning in coastal areas under future climate-change scenarios.

**Figure 1:**
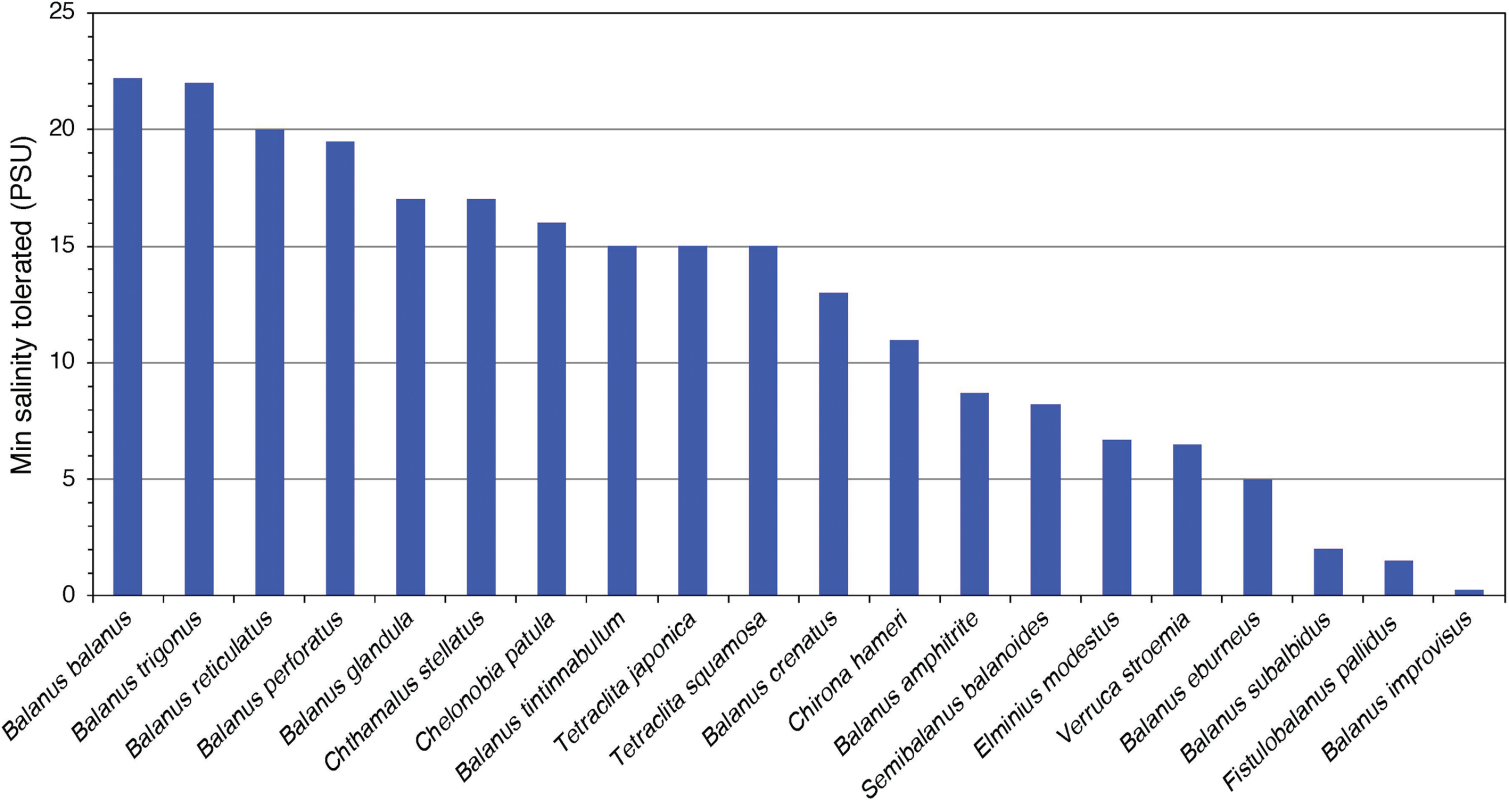
The tolerance to lowest salinities (PSU; 25 PSU ≈ 715 mOsm kg^−1^) reported from published experimental studies for 20 barnacle species (for references, see Supplementary Table S2). The included studies are very diverse in methods, life stage included, and measured response variables (for details, see Supplementary Table S1), and this overview only gives a coarse view of the interspecific variability in salinity tolerance.

During the 1960s and 1970s, several studies were published of how barnacle survival, behavior, reproduction and settlement respond to decreasing salinity (Crisp and Costlow, 1963; Barnes and Barnes, 1974; Davenport, 1976; Cawthorne, 1979). By closing their valves (shell), intertidal barnacles can cope with short-term exposure to low-salinity conditions (Foster, 1970). There are several records of barnacles tolerating low salinities either in the field or in laboratory experiments. At least 18 barnacle species can tolerate salinities below 25 PSU (Practical Salinity Unit; ≈715 mOsm kg^−1^) and at least seven species tolerate salinities even below 10 PSU (Figure 1), with tolerance measured as a range of different behavioral and physiological responses in different life stages, from embryos to adults, and during different exposure times (Supplementary Table S1). Species that stand out as particularly tolerant to low salinities are *B. improvisus*, *B. subalbidus*, *B. (Amphibalanus) eburneus*, *Verruca stroemia, B. glandula*, and *B.* (*Amphibalanus*) *amphitrite* (Figure 1). Among these, *B. improvisus* is an example of a truly brackish water species with optimal growth at low/intermediate salinities (Wrange et al., 2014). The observation of the African species *Fistulobalanus pallidus* (previously *Balanus pallidus stutsburi*) occurring in a harbor at 1.5 PSU (Sandison, 1966) may, however, require confirmation, as these observations were made in an area with highly varying salinities and 1.5 PSU was only apparent during a short time period of the year. Even though barnacles are considered to have a marine origin, there are several reports of barnacles inhabiting freshwater biotopes, e.g., the findings of active feeding and molting of *B. improvisus*, after being kept in the laboratory for more than 8 months in water of 7 mOsm kg^−1^ (<0.3 PSU), which extends the tolerance range of this species well into the freshwater region (Turpayeva and Simkina, 1961; Fyhn, 1976; Anderson 1994). It should be pointed out, however, that most studies on salinity tolerance in barnacles have not tested such extreme salinity ranges (Foster, 1970; Davenport, 1976). Furthermore, previously documented salinity tolerance has been shown to vary depending on geographical location and experimental setup, e.g., for *B. improvisus*, the minimum salinity tolerance varied from 0.3 to 9.5 PSU between different studies (Supplementary Table S1).

Despite the potentially high ecological and evolutionary importance of broad salinity tolerance in euryhaline barnacles, little is known about the mechanisms enabling them to survive and reproduce in brackish environments. Only a few studies have investigated the osmoregulatory ability of barnacles (Fyhn, 1976; Gohad et al., 2009); however, physiological and molecular information is very limited (Lind et al., 2013, 2017). The major challenge for marine invertebrates in brackish environments is to avoid swelling and to maintain functional concentrations of essential ions (Prosser, 1973; Pequeux, 1995; Withers, 1996). Euryhaline barnacles have repeatedly invaded estuaries and brackish waters and may offer mechanistic insights into parallel evolution of adaptations to a life at low salinities (Lee and Bell, 1999). Although Fyhn (1976) demonstrated an osmoregulatory capability in barnacles, nothing is yet known about the localization of the osmoregulatory functions or the cellular mechanisms of these regulations. In addition, nothing is known about the possible mechanistic diversity in osmoregulatory strategies among euryhaline barnacles and why species differ in their degree of tolerance (Figure 1).

The aim of this review is to provide an overview of the current body of literature on salinity tolerance in barnacles and to propose new ways to fill the present knowledge gap about key osmoregulatory adaptations in barnacles in dilute environments. We start the discussion with brief accounts on the concept of homeostasis and known osmoregulatory and osmoconforming strategies in other marine organisms, in particular other crustaceans, providing examples of possible mechanisms that may be involved in barnacle osmoregulation. We then turn our attention to what is currently known about these processes in barnacles, highlighting some of the recent reports that provide molecular information. We finally propose a new research agenda based on the current state-of-the-art methodologies, to improve the understanding of physiology, ecology, and evolution of osmoregulation in euryhaline barnacles.

## Homeostasis and Osmoregulatory Strategies

A prerequisite for life is to be able to maintain a stable/tolerable intracellular environment irrespective of changes in the external environment (Stott, 1981; McEwen and Wingfield, 2003). This ability of an organism is termed “homeostasis” (Cannon, 1929). Most internal variables are controlled by multiple regulatory mechanisms and the concept of “allostasis” includes the sum of all physiological and behavioral mechanisms that collectively maintain homeostasis (Korte et al., 2005). All forms of allostasis require energy, and the more severe the stress (i.e., the disturbance of homeostasis), the higher the allostatic load and the lesser the energy available for other biological functions (Moberg, 2000; McEwen and Wingfield, 2003).

**Figure 2:**
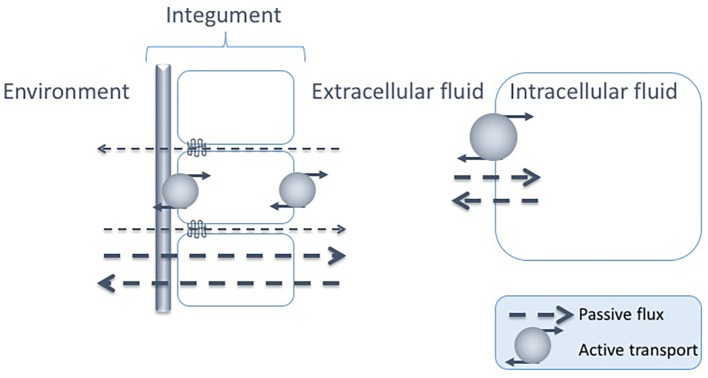
Passive fluxes of solutes occur constantly across the epithelia of marine animals, i.e., para- or transcellularly across the epithelial cell layers and across the cuticle/exoskeleton when present. There are also passive fluxes of solutes across the cell membrane. The passive fluxes are counteracted by active transports across both interfaces: the epithelia and the cell membrane, constituting two possible sites for regulated solute transport: between the intra- and extracellular fluids and between the extracellular fluid and the environment.

For animals living in aquatic environments, there is a constant passive exchange of solutes and water across all epithelia. In order to understand various strategies to maintain osmotic homeostasis, the biological fluids of an organism can be identified to belong to either of two different compartments: (1) the intracellular compartment (i.e., the fluid inside the cell membrane) and (2) the extracellular compartment, where the fluid is named coelom fluid, hemolymph, interstitial fluid or blood, depending on the organism. In line with this, passive, as well as counteracting active, exchange of solutes and water can occur across two main interfaces, between the intra- and extracellular fluid and between the extracellular fluid and the external environment (Figure 2).

**Figure 3:**
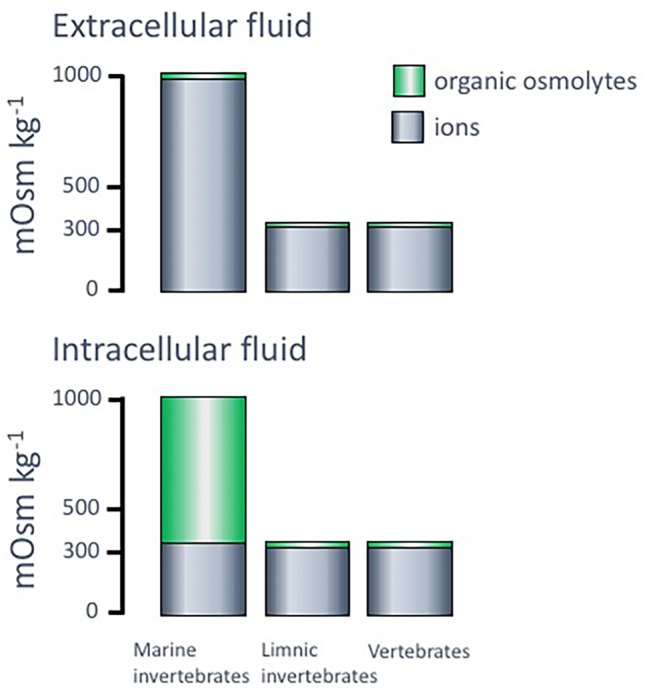
Typical levels of ions and organic osmolytes, in mOsm kg^−1^, in intra- and extracellular fluids of different organisms. Adopted from Withers (1996).

One fundamental feature regarding osmosis and ionic concentrations in most organisms is that the intracellular ion concentrations are very similar in composition and total osmolality (approximately 300–400 mOsm kg^−1^) irrespective of organism and condition. The main intracellular cation is potassium (K^+^), while both sodium (Na^+^) and calcium (Ca^2+^) are kept at low intracellular concentrations. Regarding the negative ions, main components are anionic proteins and phosphate compounds, whereas chloride (Cl^−^) is kept at low levels. Important contributors to the total osmolality in both intra- and extracellular compartments of organisms are small organic solutes like amino acids and their derivatives, polyols, sugars, methylamines, and urea (Yancey, 2005) that can vary both in the intra- and extracellular compartments (Figure 3).

Organisms in aquatic environments are traditionally referred to as osmoconformers or osmoregulators. When categorizing animals according to these definitions, the relationship between the external environment and the extracellular compartment (blood, hemolymph) is considered. An osmoconformer is an animal in which the osmolality of the extracellular fluids follows any change in the external osmolality. Thereby, osmoconformers do not waste energy on homeostasis at the extracellular level, but only for controlling the intracellular compartment. For marine osmoconformers, when experiencing an external environment of around 35 PSU (1,000 mOsm kg^−1^, full strength seawater), the difference between this osmolality that is also present in the extracellular fluid and the osmolality from the ionic parts of the intracellular fluid, equals 600–700 mOsm kg^−1^ and is termed the osmotic gap. This gap needs to be filled by an accumulation of organic solutes, termed organic osmolytes. Organisms that are osmoregulators, on the other hand, use energy and specialized osmoregulatory tissues to maintain the osmolality of the extracellular fluids at relatively stable level irrespective of the osmolality of the external environment. An organism can be a strict osmoconformer or a strict osmoregulator, but as will be discussed below, marine invertebrates frequently use a combination of the two strategies. In addition, both euryhaline (broad salinity tolerance) and stenohaline (narrow salinity tolerance) species and populations can display both strategies. Stenohaline osmoconformers also lack mechanisms to regulate their intracellular osmolyte concentrations and can therefore only tolerate very small variations in external osmolality.

## Basic Osmoregulatory Mechanisms in Marine Animals

### Main Osmoregulatory Principles

Marine invertebrates generally have extracellular fluids isoosmotic to seawater and intracellular fluids isoosmotic with the extracellular fluids, while maintaining low intracellular levels of inorganic ions. Their overall main strategy for this is to use organic osmolytes to fill the osmotic gap created between total osmolality and inorganic ion concentrations. When exposed to decreased environmental salinities, they can: (1) osmoconform by allowing water fluxes to passively alter both extracellular and intracellular osmolalities, (2) allow water fluxes to dilute the extracellular fluid but respond with intracellular volume regulation through down-regulation of the intracellular osmolytes, and/or (3) osmoregulate by active transport of ions to maintain a high osmolality in the extracellular fluids and thus become hyperosmotic to the diluted environment. Most marine invertebrates with the capacity to hyperosmoregulate in dilute environments rely on osmoconformation at higher salinities. A common way to classify marine invertebrate osmoregulators into strong, medium, or weak osmoregulators is based on this feature, where the strong osmoregulators osmoconform only in a very narrow range, or not at all, whereas weak osmoregulators may osmoconform to an osmolality that is isosmotic with the intracellular ion levels before starting hyperosmotic regulation. Thus, this classification is based on the osmotic gradients that the animals are or are not able to maintain across the body surface (environment versus hemolymph; Shaw, 1961; Mantel and Farmer, 1983; Larsen et al., 2014).

### Osmoregulatory Strategies in Crustaceans

Among marine crustaceans, all the above-mentioned strategies of vertebrates can be found. For most hyperosmoregulatory crustacean species, the mechanisms seem to be triggered only when the external osmolality reaches a certain level (approximately 26 PSU ≈ 700 mOsm or lower; see Henry et al., 2012). Thus, many euryhaline crustaceans are osmoconformers above 26 PSU, and osmoregulators at lower salinities, e.g., the green crab *Carcinus maenas* (Cieluch et al., 2004) and the blue crab *Callinectes sapidus* (Henry, 2005). From an evolutionary point of view, it is believed that osmoconformation possibly reflects the original strategy of marine crustaceans. For marine crustaceans capable of hyperosmoregulation, there seems to be an even greater diversity of both ion-absorbing mechanisms and epithelial permeabilities than has been described for hyperosmoregulatory vertebrates (i.e., freshwater fish). The recent extensive research and increased knowledge on ion-absorptive mechanisms in decapod crustacean gills have even led to the suggestion to revise the classification of strong, moderate, and weak hyperosmoregulators and instead use a terminology that distinguishes the mechanisms of ion absorption based on gills with leaky (high ion conductance) or tight (low ion conductance) epithelia (Henry et al., 2012; McNamara and Faria, 2012).

The principal adaptive strategies of osmoregulation in crustaceans during acclimation and adaptation to low salinities are: (1) reduction of the permeability of the body surface to salt and water (Kirschner, 1991), possibly by the regulation of tight junction proteins and aquaporins as shown in vertebrates (Rahi et al., 2018); (2) reduction of the osmotic gradient maintained across the body surface through osmoconformation and/or osmolyte synthesis (Gilles, 1979); (3) increased production of urine to compensate for the passive inflow of water where most euryhaline crustaceans produce urine that is isosmotic with the hemolymph, resulting in a considerable salt loss (Lockwood, 1977); and (4) active absorption of ions in order to maintain a hyperosmotic extracellular fluid when the dilution of the surrounding is extensive (Freire et al., 2008; Henry et al., 2012). Osmoregulatory mechanisms in crustaceans are thus highly diverse and are performed by the body wall, gills, and other specialized tissues, where their relative importance can vary during different developmental stages (Cieluch et al., 2004).

#### Gills

##### Mechanisms of Ion Absorption

When present, gills are the major site for active Na^+^ and Cl^−^ absorption in most osmoregulating crustaceans in low-salinity environments (Henry et al., 2012). The crustacean gill is a multifunctional organ, involved in a number of physiological processes, including gas exchange, acid-base balance regulation, and ion transport. The posterior gills act as the primary sites of active NaCl uptake in euryhaline marine crabs and the relative proportion of thick epithelium, containing ion-transporting cells (ionocytes), compared to the thin epithelium (for gas exchange), increases when exposed to diluted environments (Lovett et al., 2006). Interestingly, osmoregulatory crustaceans have evolved considerably different mechanisms for active NaCl absorption and display marked differences in the electrical conductance of their gill epithelia compared to vertebrates (Henry et al., 2012). Most transporters found in gills of hyperosmoregulatory decapods are similar to those described for freshwater fish, but the transporter repertoire in crustaceans appears to be divided into two main groups of transporters which are expressed in relation to the tightness of the paracellular pathway of the gill epithelium (Henry et al., 2012).

**Figure 4:**
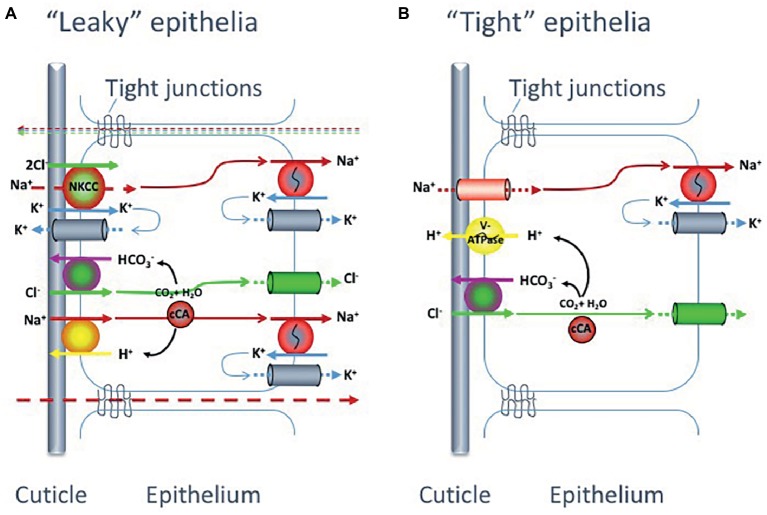
Proposed models for monovalent ion uptake across the gills of hyperosmoregulating decapod crustaceans, according to Henry et al. (2012). Experimental data suggest that crabs possess one of two main gill epithelial types: **(A)** with low transepithelial potential, indicating high paracellular permeability and thus a “leaky” epithelia or **(B)** with high transepithelial potential suggesting low paracellular permeability and a “tight” epithelia. The two suggested epithelial types have partly different transport and channel protein repertoires.

Species with “leaky” tight junctions, i.e., epithelial paracellular pathways with high permeability, have problems maintaining high electrochemical gradients and will have to generate high rates of active ion absorption to compensate for this leakage. These organisms invest considerable amounts of energy in active uptake of ions (Henry et al., 2012). Species with “tight” epithelia, on the other hand, can more easily maintain a high electrochemical gradient without high transport rates, hence spending less energy on hyperosmoregulation (Figure 4).

Even though the two types of gill epithelia (“tight” and “leaky”) in hyperosmoregulatory crustaceans reveal different ion transporter and channel repertoires (Figure 4), both systems are equipped with three transporters in common: the Na^+^/K^+^-ATPase, the K^+^ channel and the Cl^−^ channel. The details of the differences in ion transporters and related pathways between “tight” and “leaky” epithelia in crustaceans have recently been described by Henry et al. (2012) and will only be briefly summarized here. As for the vertebrate hyperosmoregulators, the Na^+^/K^+^-ATPase in crustaceans is basolaterally localized and functions as the main driving force for the NaCl absorption at low salinity in both epithelial types.

In the “tight” epithelium, the Na^+^ gradient generated by the Na^+^/K^+^-ATPase allows for Na^+^ absorption across the apical membrane through a Na^+^ channel (ENAC) that is electrogenically coupled to adjacent apical V-type H^+^-ATPases. This primary active transport of H^+^ ions also aids in the active absorption of Cl^−^ ions by providing HCO_3_^−^ to apical Cl^−^/HCO_3_^−^ exchangers, as the H^+^-ATPase together with a cytoplasmic carbonicanhydrase (CA) increase intracellular levels of HCO_3_^−^. The Cl^−^ absorption proceeds to the hemolymph *via* basolateral Cl^−^ channels while the absorbed Na^+^ is secreted out from the cell through the Na^+^/K^+^-ATPase.

For the “leaky” epithelia, the situation is more complex. In this epithelial model, the Na^+^ and Cl^−^ uptake is directly coupled and there is no apical V-type H^+^-ATPase present. The basolateral Na^+^/K^+^-ATPase builds the inwardly directed Na^+^ gradient, which drives Cl^−^ into the cell *via* a Na^+^-K^+^-Cl^−^ cotransporter (NKCC) in the apical membrane. Absorbed Na^+^ ions are then pumped across the basolateral membrane by the Na^+^/K^+^-ATPase. The apical K^+^ channels are important to supply the NKCC with K^+^ ions as well as for maintaining a negative inner potential of the cell. The Cl^−^ will thereby move along its electrochemical gradient to the hemolymph through Cl^−^ channels in the basolateral membrane. In addition, active Na^+^ and Cl^−^ uptake proceeds *via* cation, Na^+^/H^+^ (NHE), and anion, Cl^−^/HCO_3_^−^, exchangers in the apical membrane supported by a cytoplasmic carbonic anhydrase (CA) that catalyzes the production of acid/base equivalents as substrates for the exchangers.

##### Epithelial Permeability

For marine crustaceans, very little is presently known about the molecular mechanisms behind the differences in paracellular permeabilities, but differential expression of different tight junction proteins is a probable mechanism. The tight junctions consist of several physiologically regulated proteins forming the circumferential seals around adjacent epithelial cells. Three of the main protein families found in the tight junctions are occludins, claudins, and junction-associated membrane proteins (JAM). The claudins and occludins form the backbone of the tight junction, and the type of claudins expressed as well as the number of occludin strands are suggested to determine the epithelial permeability (Schneeberger and Lynch, 2004; Van Itallie et al., 2008). The different claudin isoforms display different number and types of charged amino acid residues lining the pore that is formed between the adjacent cells, which constitute the passage for molecules using the paracellular pathway. The differential expression of claudin isoforms has therefore been suggested to be the main determiner of tight junction ion and size selectivity (Van Itallie and Anderson, 2006; Amasheh et al., 2011).

Not only the paracellular, but also the transcellular pathway may constitute an important route for movement of ions and water across an epithelium (Sundell and Sundh, 2012). The transcellular permeability for water is set by the permeability of the lipid bilayer and by the presence in the lipid bilayer of transmembrane aquaporins (AQPs). AQPs are a family of channel proteins permeable to water and various small molecules (Finn and Cerda, 2015), where some isoforms are differentially expressed in response to salinity in the intestine of migrating fish (Kim et al., 2010; Tipsmark et al., 2010; Sundell and Sundh, 2012). One AQP has been isolated and characterized from the euryhaline blue crab (*C. sapidus*), and this AQP was induced during low-salinity conditions and showed tissue-specific expression (Chung et al., 2012). Genome-based identification and functional characterization was recently performed on the AQPs from the salmon louse, *Lepeophtheirus salmonis*, a marine ectoparasitic copepod that feeds on the skin and body fluids of salmonids (Stavang et al., 2015). Phylogenetic analysis of the seven AQP paralogs identified in the genome of *L. salmonis* indicated that two belong to classical AQPs (encompassing the water-transporting AQPs), three are aquaglyceroporins (that besides water transport glycerol), and two are unorthodox AQPs (with unknown function). Functional assays reveal that the permeation properties of the different crustacean AQPs are largely conserved to their vertebrate orthologs. Transcript analyses revealed expression of all except one of the louse AQPs at all developmental stages; however, potential expression changes in response to different salinities were not examined (Stavang et al., 2015). Transcriptome data from another salmon louse, *Caligus rogercresseyi,* also revealed a similar repertoire of expressed AQPs (Farlora et al., 2014).

#### Antennal Glands

The antennal glands (the ultra-filtrating kidney-equivalent in marine crustaceans) are not particularly important for osmoregulation under isosmotic circumstances. During hypoosmotic exposure, on the other hand, the excretion of excess water is accomplished through filtration of large amounts of urine. However, most euryhaline crustaceans are not able to produce hypoosmotic urine (like some freshwater species can; Riegel, 1968). Instead, they produce urine that is isosmotic with the hemolymph, resulting in concomitant salt loss.

#### Shell-Closing Mechanisms

Animals with well-defined and closable exoskeletons, like gastropods and bivalves among the molluscs, or barnacles among the crustaceans, can offer a rapid behavioral escape response to shorter intervals of salinity changes. By closing their shells, they can maintain a hyperosmotic inner environment (the mantle cavity) in relation to a diluted external environment. It was found in the periwinkle snail *Littorina littorea* that the rate of salt and water exchange between the mantle cavity (extra-visceral cavity, inside the shell but outside the body), and the ambient water is rather slow, and even after 12 days of freshwater exposure, the osmolarity in this cavity was only reduced by roughly 25% (Sokolova et al., 2000). Similar results have been found for the blue mussel *Mytilus edulis* (Davenport, 1979).

## Osmoregulation in Barnacles

### Valve Closure and Osmotic Evasion

Acorn barnacles (Sessilia) display an evasive behavior by withdrawing the cirri and prosoma and closing the opercular valves for long periods of time, when encountering adverse environmental conditions, e.g., freshwater (Barnes et al., 1963; Cawthorne, 1979). This enables them to survive in the harsh environment of the splash zone, where they are regularly exposed to long periods of desiccation or immersion in freshwater during rainfall. The effect of opercular closure can be exemplified by species in the genera *Balanus* and *Elminius,* which needed up to 7 days of incubation to reach a new osmotic steady state when transferred directly from 100 to 50% seawater (Foster, 1970). Closing the valves is, however, not a permanent solution to low-salinity conditions; the internal salts are ultimately lost, and during closure, the barnacle cannot perform vital activities like feeding and respiration. As soon as beating of the cirri and the movements of the prosoma are regained, it will produce a flow of external medium in and out of the mantle cavity to fulfill respiratory, nutrient absorption, and excretory needs, leading to exposure to the dilute medium and the need for an osmoregulatory strategy.

### Evidence of Various Modes of Osmoregulation in Barnacles

When immersed in low salinities for longer times, barnacles may either act as osmoconformers or osmoregulators. Only a few studies have investigated the osmoregulatory ability of barnacles at lower salinities and the results are somewhat complex. Early work by Belyaev (1949), using freezing point depression assessment of the hemolymph osmolality, suggested hyperosmotic regulation in, e.g., *Semibalanus balanoides*, and Newman (1967) found a similar maintenance of a hyperosmotic hemolymph in *B. amphitrite* but not in *B. improvisus*, which instead conformed with a reduction in salinity. These results of apparent osmoregulation were challenged by Foster (1970) from studies of osmolality of both mantle cavity fluid and hemolymph in several barnacle species. Foster concluded that the maintenance of a hyperosmotic hemolymph in low external salinities was only short-term and mainly an effect of valve closure, enabling a maintained hyperosmotic mantle fluid and thereby a hyperosmostic hemolymph. During long-time exposure (>24 h) to reduced salinity, which also involved valve opening, the barnacle species studied reached isoosmosity of the hemolymph to the diluted environment. Foster (1970) therefore suggested that active, long-term acclimated barnacles should be considered osmoconformers.

**Figure 5:**
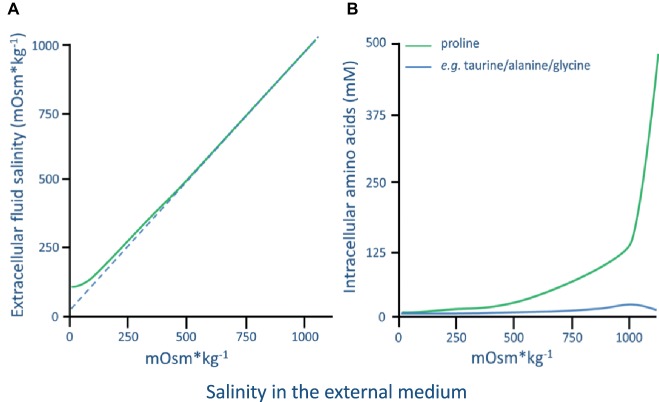
**(A)** The hemolymph/extracellular fluid osmolality in *Balanus improvisus* acclimated to a range of ambient external salinities. **(B)** Concentration (mM) of free amino acids in the thorax muscle of *B. improvisus* acclimated to a range of ambient external salinities. 1,000 mOsm kg^−1^ is approximately 35 PSU (graphs are adapted from Fyhn, 1976).

In the most extensive and long-term study of a euryhaline barnacle, Fyhn (1976) investigated the osmolality and chloride ion concentrations in the mantle cavity fluid, maxillary gland fluid, and hemolymph of *B. improvisus*. In fully acclimated adult animals (acclimated for up to 5–10 weeks), it was found that *B. improvisus* started to exhibit hyperosmotic regulation of the hemolymph in salinities below 500 mOsm kg^−1^ (17 PSU). Below 100 mOsm kg^−1^ (approx. 3 PSU), the hemolymph osmolality was kept stable at 100 mOsm kg^−1^ demonstrating a hyperosmoregulatory ability efficient enough to maintain an osmotic gradient of 15:1 across the body surface at environmental salinities of approximately 7 mOsm kg^−1^ (Figure 5A; Fyhn, 1976). In salinities above 500 mOsm kg^−1^, *B. improvisus* was demonstrated to fully osmoconform and exhibit intracellular volume regulation mainly by the aid of the amino acid proline (Figure 5B; Fyhn, 1976). However, it is clear that barnacle species behave differently to low salinities. While *B. improvisus* partly osmoconforms down to salinities of about 100 mOsm kg^−1^, other barnacles like *E. modestus* and *S. balanoides* hyperosmoregulate at around 800 mOsm kg^−1^ (Foster, 1970). Thus, *B. improvisus* is able to show cirral activity and to maintain the hemolymph at an osmolality as low as 100 mOsm kg^−1^ through hyperosmoregulation, whereas many other barnacles appear to start hyperosmoregulation at osmolalities that are at least 5× higher (Foster, 1970). As described above, most organisms exhibit an intracellular osmolality based on ion concentrations of around 300 mOsm kg^−1^, making *B. improvisus* relatively extreme in its ability to maintain vital metabolic functions at very low intracellular ionic concentrations (100 mOsm kg^−1^).

### Active Ion Transport in Barnacles

Over 40 years ago, active ion transport was hypothesized to be the primary mechanism for osmoregulation in adult barnacles at low salinities (Fyhn, 1976). However, there are yet no experimental data that firmly establish a primary tissue or site for ion pumping used for barnacle osmoregulation. The mantle epithelium in adult barnacles has been proposed as the primary gas exchange and salt excretion organ (Anderson and Southward, 1987). Gohad et al. (2009) showed that *B. amphitrite* has a mantle epithelium that is rich in cells that stained intensively with silver nitrite (forming a black silver chloride precipitate that indicates cells rich in chloride ions) and that produces an electrolyte-rich secretion. Silver chloride-stained epithelia were also found in barnacle larval stages (nauplius and cyprid) and were shown to contain mitochondria-rich cells. Although the study did not include exposure to low salinities, the authors speculate that these cells might be involved in osmoregulation at low salinities (Gohad et al., 2009). In line with this study, *B. improvisus* hemolymph chloride ion concentrations were up-regulated when exposed to seawater below 100 mOsm kg^−1^, suggesting that the Cl^−^ is one of the main monovalent ions to be up-regulated in order to maintain a hyperosmotic state in dilute environments (Fyhn, 1976). In neither *B. amphitrite* nor *B. improvisus*, the hemolymph Na^+^ concentrations were measured, but a similar up-regulation of hemolymph Na^+^ concentrations can be expected assuming an osmoregulatory mechanism actively absorbing NaCl similar to what has been shown for other osmoregulatory crustaceans (see above). The mantle provides a large surface area and is the tissue first in contact with the surrounding water, making this tissue a plausible site for osmoregulation in barnacles. Barnacles also have “branchiae” that are attached to the mantle and have been suggested to function as gills (White and Walker, 1981; Gohad et al., 2009). Since gills are considered the principal sites for maintaining salt balance in osmoregulating crustaceans (see above), the mantle along with these branchiae may provide the extensive surface needed for active ion transport during osmoregulation. This has, however, yet to be thoroughly investigated.

As described above, the primary ion transporter, Na^+^/K^+^-ATPase, is the main driving force in osmoregulation when marine organisms are challenged with changing salinities, e.g., both the activity and the expression of the Na^+^/K^+^-ATPase were enhanced in the blue crab *Callinectes ornatus* during acclimation to low salinity (Leone et al., 2015). Recently, it was established that *B. improvisus* has two main gene variants of Na^+^/K^+^-ATPases, *NAK1* and *NAK2*, which are roughly 70% identical at the protein level (Lind et al., 2013). In addition, the authors showed that the *NAK1* mRNA existed in a long and a short isoform with the encoded proteins differing only by 27 N-terminal amino acids. This 27 amino acids-long N-terminal stretch was encoded by a separate exon, and the two variants of *NAK1* mRNAs are created by alternative splicing. Interestingly, the two *NAK1* isoforms were differentially expressed in response to salinity. When exposed to low salinity, the expression of the long *NAK1* mRNA increased relative to the short. It was thus suggested that the alternatively spliced long variant of the Nak1 protein might be of importance for osmoregulation in *B. improvisus* in low-salinity conditions. However, at this stage, nothing is known about the location of the Nak1 and Nak2 proteins, or about the alternatively spliced versions of *NAK1*. This important transporter in osmoregulation has not been studied in other barnacle species.

### Osmoregulation and Excretion Organs

The antennal and/or maxillary glands of crustaceans are the excretory organs used to produce and excrete urine (White, 1987). They consist of three main regions – the end sac, the excretory canal, and the short efferent duct (White and Walker, 1981). Darwin (1851) first described the end sac complex in the anterior body of barnacles although an excretory function was not suggested until later (reviewed in Nilsson-Cantell, 1921). The end sac of the maxillary gland of adult barnacles consists of podocyte-like cells that have marked morphological and cytochemical similarities to the equivalent region of other Crustacea and to the vertebrate glomerulus (White and Walker, 1981). The striking similarity of such cells within animals of differing phylogeny indicates that the basic mechanism of primary urine formation is likely to be similar. In the barnacle *B. improvisus*, maxillary gland fluid is isoosmotic to the hemolymph (Fyhn, 1976). Thus, the maxillary glands in this species do not seem to be involved in the active osmotic regulation of the hemolymph. Barnacles seem to be similar to marine and brackish water decapod crustaceans where isoosmocity and chloride isoionicity of urine to hemolymph are found (Schoffeniels and Gilles, 1970).

### Osmoregulation and Aquaporins

As mentioned previously, aquaporins are a family of channel proteins that are permeable to water and small molecules and play important functional roles in urine formation as well as for generally setting the epithelial permeability. Based on genome and transcriptome sequencing, the repertoire of eight AQPs from the barnacle *B. improvisus*, was recently presented (Lind et al., 2017). Phylogenetic analysis revealed that this barnacle has members of the classical water AQPs (Aqp1, Aqp2), the aquaglyceroporins (Glp1, Glp2), the unorthodox AQP (Aqp12), and the arthropod-specific big brain aquaporin (Bib). Interestingly, two big brain-like proteins (BibL1 and BibL2) were also found, constituting a totally new group of AQPs not yet described in arthropods. In addition, two of the water-specific AQPs were expressed as C-terminal splice variants; thus *B. improvisus* express in total 10 variants of AQPs. Functional characterization showed that Aqp1 transports water and Glp2 water and glycerol, agreeing with the predictions based on 3D modeling and phylogeny. The functional role(s) of Bib and the BIB-like proteins in barnacles are currently not known. A possible role for the *B. improvisus* AQPs in osmoregulation was found from mRNA expression changes of some of the AQPs in adult barnacles analyzed after long-term acclimation to different salinities. In particular, *AQP1* displayed the most pronounced expression difference with a substantial (>100-fold) decrease in the mantle tissue at low salinity (3 PSU) compared to high salinity (33 PSU). This low salinity-triggered repression might be a mechanism to reduce the osmotically driven transcellular uptake of water at the large surface area of the mantle in low-salt environments, and thus be an important factor for this species’ invasive success in brackish/freshwater conditions.

### Osmolyte Systems

Among the amino acids, proline is probably the most widely distributed osmolyte, and the accumulation of this amino acid has been observed in a wide array of organisms (Yancey et al., 1982). In hypersaline conditions, proline has been shown to dominate the intracellular amino acid pool in several barnacles. In *B. improvisus*, e.g., proline constitutes 86% of the total intracellular concentration of amino acids (Fyhn, 1974, 1976), while in the barnacle *S. balanoides*, it reaches levels up to 53% (Cook et al., 1972). In the case of *B. improvisus*, intracellular volume regulation is achieved through a proline concentration increase of about 2,000 times when acclimated from low (7 mOsm kg^−1^) to high (1,000 mOsm kg^−1^) salinity (Figure 5B; Fyhn, 1976). *B. improvisus* thus shows a high utilization of proline in the intracellular volume regulation and proline metabolism is probably a key factor for the euryhalinity of this species. In decapod crustaceans, it has been shown *in vitro* that the activity of various enzymes involved in amino acid metabolism is dependent upon the concentration of inorganic ions, thus suggesting a control of the cellular amino acid pool by the concentration of inorganic ions in the cytoplasm (see review Schoffeniels, 1976). The pathway from glutamate is the primary route for the synthesis of proline under conditions of osmotic stress, and in the blue crab (*C. sapidus*) inorganic ions have been shown to regulate *de novo* synthesis of proteins, presumably enzymes catalyzing this pathway, leading to a targeted *de novo* synthesis of proline during hyperosmotic stress (Burton, 1992). It has not been established if this also could be a mechanism for enzyme activation in barnacles.

**Figure 6:**
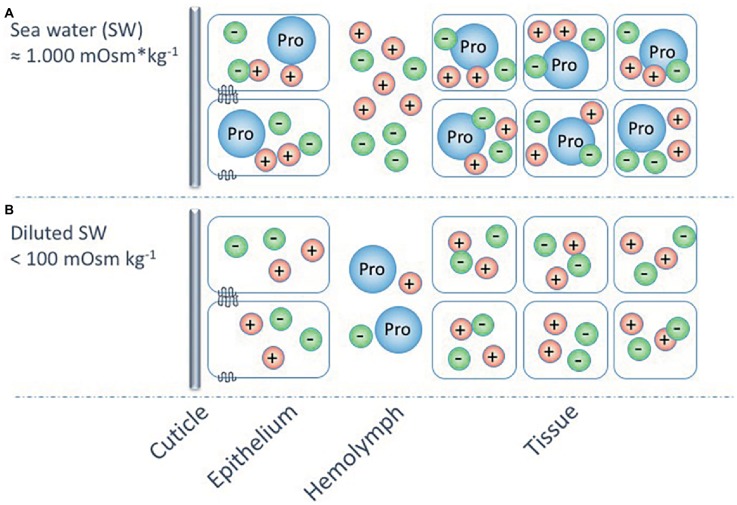
A hypothetic model (partly based on Fyhn, 1976) for the osmoregulatory strategies of the euryhaline barnacle, *Balanus improvisus*, at different external salinities. The model shows osmoconformation and intracellular volume regulation, using mainly the amino acid proline (Pro), at salinities around 1,000 mOsm kg^−1^ **(A)**, and hyperosmoregulation, using active ion transport in combination with proline as extracellular osmolyte, at salinities below 100 mOsm kg^−1^ **(B)**. The possible use of proline as an extracellular osmolyte at these extremely low environmental osmolalities may be a mechanism for increasing the hemolymph osmolality, while preserving available ions for the intracellular milieu.

For the barnacle *B. improvisus*, proline has not only been suggested as the most important intracellular osmolyte in seawater, but Fyhn (1976) also proposed that this amino acid functions as an extracellular osmolyte adding to the maintenance of a hyperosmotic hemolymph at low environmental osmolalities. This suggestion emanates from the findings that the proportion of chloride ions, in relation to total hemolymph osmolality, seemed higher when the barnacles were kept in high salinities (in their osmoconforming state) than during hyperosmotic regulation. The Cl^−^ concentrations constituted about 50% of the hemolymph osmolality in seawater but only about 30% in animals kept in media below 100 mOsm kg^−1^ (Fyhn, 1976). Together with its cationic counter-ions, mainly Na^+^, these ions would make up the bulk of the hemolymph osmolality at high salinities but only about 60% of it in the osmoregulatory state. Thus, Fyhn (1976) suggested that proline most probably constitutes about 40% of the hemolymph osmolality in low salinities. A working hypothesis could be that euryhaline barnacles can hyperregulate the hemolymph at low salinities, not only by active uptake of monovalent ions but also by producing and excreting proline, or other osmolytes, from surrounding cells (Figure 6). This could suggest an “ion-saving” mechanism to ensure that most ions are used intracellularly for proper metabolic cell functions or it could reflect a low ion-absorbing capacity of *B. improvisus* that instead adopts the strategy to maintain a hyperosmotic hemolymph by the aid of organic osmolytes.

## Evolutionary Consequences of Osmoregulatory Mechanisms

### Osmoregulation and Exploitation of Novel Habitats

Estuaries are characterized by fluctuations in physical and chemical conditions, resulting in a harsh environment where special physiological adaptations are required to survive and reproduce. The challenge for organisms living in estuaries is reflected by the low species diversity in intermediate and fluctuating salinities, compared to the situation in freshwater and marine environments (Remane and Schlieper, 1971; Cognetti and Maltagliati, 2000). However, lower species richness also means that there are potentially more available ecological niches, which can be utilized by species that physiologically can cope with the environmental conditions, like low salinity (e.g., Paavola et al., 2005). Furthermore, estuaries often have certain advantages such as being nutrient rich (Mann and Lazier, 2005), thus providing food resources that may compensate for stressful salinity conditions. Lee et al. (2013) showed that high food concentrations increased low-salinity tolerance in the euryhaline copepod *Eurytemora affinis*, enabling invasion of this species in brackish and freshwater environments.

Successful invasions in brackish environments are also often associated with the lack of natural predators, competitors, and pathogens, which are not able to cope with the harsh estuarine conditions (i.e., the “enemy release hypothesis,” e.g., Williamson, 1996). Biological invasions have increased dramatically over the past decades due to increased coastal anthropogenic activities, including shipping, aquaculture, and indirect transports on floating marine litter. Many invasions result in major ecological and economic impact on coastal areas (Pimentel, 2005). Interestingly, some of the most widespread barnacle species have in common that they are fouling organisms on ships as well as having a broad salinity tolerance, indicating that the ability to osmoregulate plays an important role in successful invasions (Capps et al., 2011).

**Figure 7:**
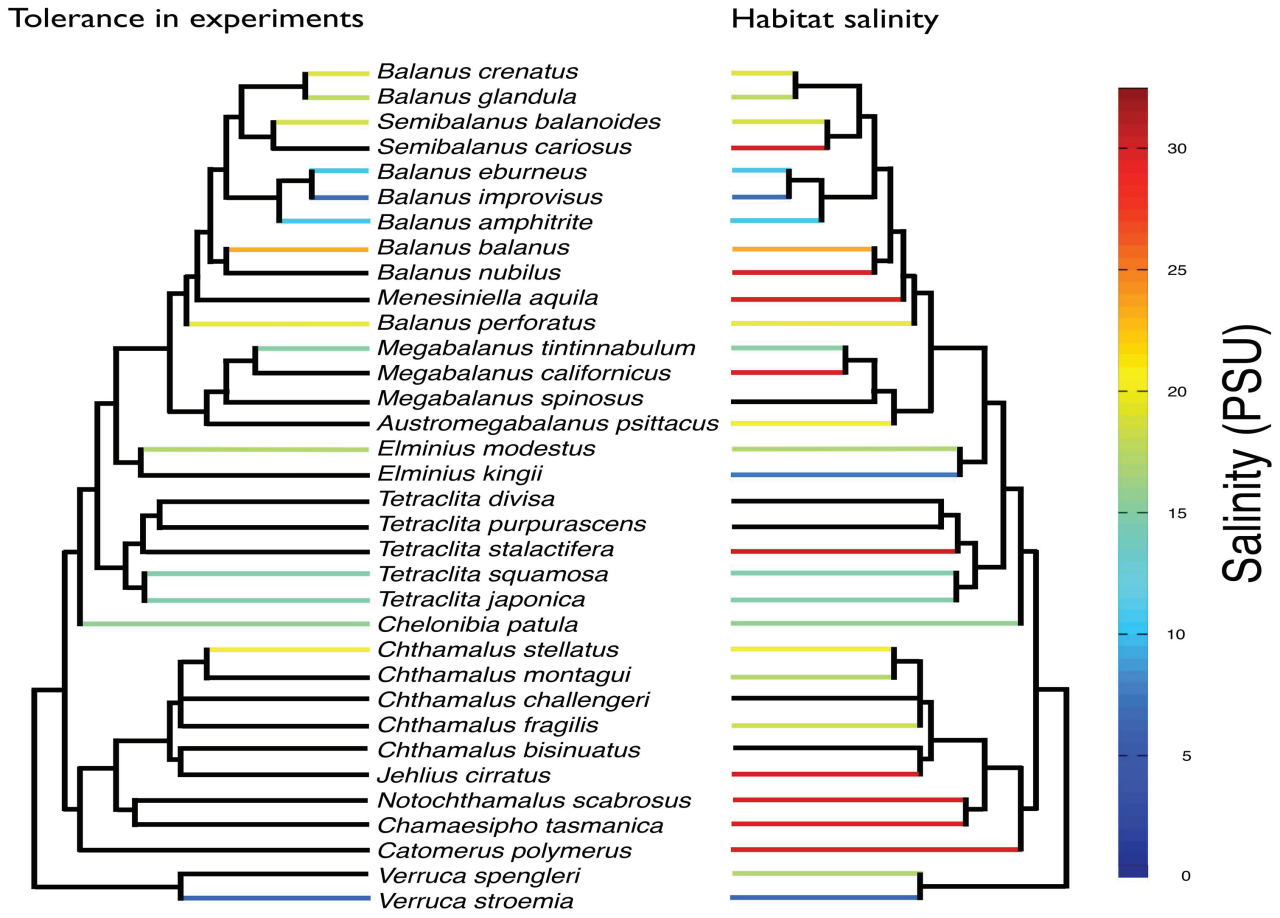
Overview of tolerance to low salinity for 15 barnacle species from experimental studies (see Supplementary Table S2 for references) mapped on a phylogenetic tree of 34 barnacle species. Also shown (right tree) is available information of lower bounds of salinity encountered in the habitats of the different species. Black branches indicate that no information was found. The phylogenetic tree was based on 18S obtained from Pérez-Losada et al. (2004) and *Balanus improvisus* (accession number MK625481). The tree was constructed with PhyML-aLRT (Anisimova and Gascuel, 2006).

Euryhalinity has also been suggested to play an important role in population divergence. Since euryhaline species can invade a broad range of habitats, e.g., along environmental gradients where different selection pressures are important, this may lead to the evolution of local adaptations and even speciation (Johannesson et al., 2011). Furthermore, brackish water bodies are often partially geographically isolated from each other, resulting in restricted gene flow between estuaries and brackish seas, also promoting population differentiation (Bilton et al., 2002). Selective tidal stream transport (e.g., Morgan, 1995) in estuaries also provides potential for high retention of larvae within estuaries, restricting gene flow and promoting differentiation between populations that reside in such areas. There are several examples where intraspecific divergence in, e.g., morphology, physiology, behavior, and life history traits have evolved between populations in freshwater, brackish, and marine environments (Bilton et al., 2002). Barnacles that have been experimentally shown to tolerate low salinities are also generally found in habitats with reduced salinity (Figure 7). Some species are rather extreme in their euryhalinity; e.g., *B. improvisus* display strong phenotypic plasticity with respect to salinity (Wrange et al., 2014; Nasrolahi et al., 2016). The most prominent euryhaline barnacles form a phylogenetic clade, although there are also more phylogenetically distant species/groups that show tolerance to low salinities, indicating that low-salinity tolerance may potentially have evolved multiple times (Figure 7). Previous studies have also revealed evolutionary shifts associated with freshwater invasions, where increased freshwater tolerance was associated with loss in high-salinity tolerance, indicating evolutionary trade-offs in osmoregulatory traits (Lee et al., 2003, 2007), although such costs have been difficult to quantify.

### Importance of Osmoregulation for Life-History Evolution

Barnacles have pelagic larvae with limited capacity to control their dispersal, potentially leading to exposure to variations in osmotic stress in coastal and estuarine areas (Anger, 2003). Osmotic stress can strongly affect larval survival and fitness, and this selection pressure has possibly led to evolution of distinct life-history strategies and physiological adaptations in both adults and early developmental stages. There are many examples of invertebrate species where adults are more tolerant to low salinities compared to larval stages; e.g., *Carcinus maenas* larvae are not as tolerant to low salinity (>20 PSU) as adults (>4 PSU) (Anger et al., 1998). This limited larval tolerance to low salinities has been suggested to be a restraining factor in the ability of *C. maenas* to become established in brackish habitats (Bravo et al., 2007). Several strategies have evolved to overcome the problem of different tolerance between life stages (Torres et al., 2011). In the crab *Eriocheir sinensis*, adults migrate out to higher salinities to reproduce, and juveniles will later re-colonize freshwater areas (Anger, 1991). Barnacles, in contrast to most crustaceans, have sessile adult stages, requiring that the free-swimming larvae can tolerate similar salinity ranges as adults (e.g., Nasrolahi et al., 2016). Lind et al. (2013) recently showed that adult barnacles and cyprid larvae (*B. improvisus*) showed differential expression of alternatively spliced forms of Na^+^/K^+^-ATPase genes, indicating that there may be differences in osmoregulatory strategies between life stages also in barnacles.

### Osmoregulatory Mechanisms With Multiple Functions

Evolution of efficient osmoregulation may not only involve trade-offs with other fitness-related traits (e.g., reproduction or growth) but may also positively serve multiple functions. Living in the intertidal zone, barnacles are exposed to a range of environmental stressors including fluctuations in salinity but also heat, desiccation, and freezing. Different physiological adaptations have evolved to cope with such extreme conditions. Both desiccation and freezing involve withdrawing water from the tissues, thus increasing the solute concentration in the body fluids, involving mechanisms which could be connected to osmoregulation. Cook and Lewis (1971) reported that barnacles (*S. balanoides*) acclimated to desiccation also increased their cold tolerance, although the physiological mechanisms were not described. Furthermore, many invertebrates inhabiting marine waters seem more tolerant to freezing compared to brackish water species (Aarset, 1982). Changes in freezing tolerance resulting from salinity acclimation are probably related to changes in osmolyte concentration in the intracellular fluids, e.g., proline accumulation in the barnacle *B. improvisus* after high salinity exposure (Fyhn, 1976). Feeding *Drosophila melanogaster* larvae with proline-rich diet also resulted in higher freeze tolerance (Kostal et al., 2012). Hence, proline is an active drought- and freeze-resistance agent that might play an important multifunctional role in barnacles during longer periods of desiccation or freezing. It has also been suggested that aquaglyceroporins could be functional in antifreeze mechanisms for the sessile adult stage that probably needs to increase and control glycerol content in various tissues during winter to avoid freezing (Cook et al., 1972). The relative abundance of aquaglyceroporins compared to water-specific AQPs was reported higher in the adult compared to cyprids in *B. improvisus* (Lind et al., 2017), perhaps indicating that glycerol transport is of greater importance for adults. It has also been suggested that ion transporters involved in osmoregulation may aid in pH regulation also in invertebrates (Hu et al., 2011), although Lind et al. (2013) could not find any significant change in gene regulation of Na^+^/K^+^-ATPases when *B. improvisus* larvae were exposed to low pH.

### Osmoregulation and Future Environmental Change

As part of future climate-related environmental changes, salinity is predicted to decrease in many coastal areas due to increased precipitation and enhanced freshwater run-off (Boyer et al., 2005; Dore, 2005; Meier et al., 2012). As stated previously, salinity plays an important role in shaping the distribution of marine species and future alterations in salinity will pose major ecological challenges to organisms inhabiting coastal areas (Levinton et al., 2011; Wikner and Andersson, 2012); it will certainly define which species will dominate coastal communities in the future. Apart from salinity changes, other factors such as global warming, ocean acidification, and increased pollution are predicted to influence coastal ecosystems dramatically in the near future (Halpern et al., 2008). Little is, however, known about how osmoregulatory functions are influenced by other stressors, e.g., temperature and pH. There could be synergistic effects acting to reduce salinity tolerance at high temperatures, as has been observed between salinity and temperature in marine isopods (Vlasblom et al., 1977). A crucial question is if evolutionary changes in osmoregulatory functions may track/match the rate of climate change. Future studies using a combination of physiological mechanistic studies, molecular genomics, quantitative genetics, and evolution experiments may address this question (Munday et al., 2013), where barnacles would provide an excellent model.

## Future Research Directions on Barnacle Osmoregulation

There is still a lot to be learned about the physiological and molecular mechanisms behind osmoregulatory strategies in barnacles, and in particular these mechanisms’ ecological significance and evolutionary history. In this pursuit, there is a need for the involvements of experts in many diverse fields, from technology developers to theoreticians in systems biology, to further increase our understanding of how the various osmoregulatory components act in complex mechanistic and regulatory networks.

Future research on osmotic and ionic regulatory physiology in barnacles will surely benefit from a phylogenetic perspective, which has to some extent already been integrated in recent studies on other crustaceans (e.g., McNamara and Faria, 2012). A number of genome sequencing projects of various species of barnacles are underway, which open up for comparative genomics studies on osmoregulation. Comparing barnacle species with various ranges of salinity tolerance will be instrumental to provide a complete picture of the species-specific gene repertoires. Identifying candidate genes that may be involved in osmoregulatory functions will guide our understanding on how barnacles mechanistically cope with changes in salinity, in line with what has been achieved in decapod crustaceans (Henry et al., 2012; McNamara and Faria, 2012). These analyses will in particular provide a view on how osmoregulatory traits may have arisen and been shaped during evolution. The genome projects also open up for applications of various types of genome-wide methodologies in functional genomics. Maybe the most straightforward is genome-wide gene expression studies, which could be conducted on individuals acclimated or adapted to low-salinity conditions. These studies could be applied to examine mechanisms in osmoregulation in barnacles, both within and between species. Genome-wide transcriptome studies using RNA-seq have successfully been used in the blue crab, identifying well-characterized genes in osmoregulation as well as proving expression changes of genes not earlier identified to respond to low salinity, e.g., a degenerin-like sodium channel, a potassium channel, and the neuropeptide Y receptor (the latter potentially indicating mechanisms for hormonal control of osmoregulation) (Havird et al., 2016).

However, there is still a lot of functional information that gene sequences and bioinformatics analyses cannot provide. In particular, there is a knowledge-gap between sequence variation in proteins and the understanding of the functional consequences. Many of the osmoregulatory components are membrane proteins (e.g., ion transporters and aquaporins), making them hard to study from a 3D structural/functional perspective, despite the rapid progress in the structural biology field (Caffrey et al., 2012). There is therefore an urgent need to develop various manipulative methodologies in functional genomics in combination with physiological studies in the field of barnacle biology. This will certainly be a long and hard process, but the option of being able to make “knock-downs” or “knock-outs” in these systems, e.g., *via* RNAi (Zhang et al., 2015) or CRISPR-cas9 technology (Gandhi et al., 2018), would indeed prove to be worth the efforts to enable hypothesis testing. In parallel to this, we need to establish rational strategies for functional characterization of barnacle osmoregulatory components in experimentally amenable heterologous systems like *E. coli* or yeast. Expression of genes and functional characterization is today becoming an important tool in medical science where large number of genes and gene variants can be rapidly tested in heterologous systems (Mayfield et al., 2012), an activity that has been termed “surrogate genetics.” Similarly, surrogate genetics should be exploited for all the osmoregulatory components of barnacles; e.g., like sequence variants of Na^+^/K^+^-ATPase from vertebrates have been functionally characterized in yeast (Horowitz et al., 1990).

Functional studies should also include enzyme biochemistry. In this context, it is interesting to note that the Na^+^/K^+^-ATPase also has the capacity to transport ammonium instead of potassium (hydrated NH4^+^ and K^+^ ions have similar ionic radius). This results in Na^+^ uptake at low salinities also influencing the ammonia excretion and thus general metabolism, as shown for crabs (Mangum et al., 1976; Weihrauch et al., 2002; Gradinaru and Apell, 2015). The presence of paralogs of the Na^+^/K^+^-ATPase transporter in *B. improvisus* (Lind et al., 2013) opens up for studies regarding paralog-specific affinities for the counter-ions K^+^ and NH4^+^. It would also be interesting to conduct more general physiological studies on the ammonium metabolism and its role in osmoregulation in barnacles. In addition, electrophysiological studies on isolated cells, epithelial cell lines, or intact animal tissues should be conducted, but is a challenge for many of the barnacle species considering their small size. However, patch clamp techniques on isolated patches of specific membranes could be a viable experimental avenue, in particular if linked to the use of specific pharmacological tools for transport and channel proteins (Pedersen et al., 2000). We also need to develop methodologies to locate sites of expression of specific osmoregulatory genes in whole animals. The procedure of *in situ* hybridization was reported to work for both cyprids and adults of *B. amphitrite* (Dreanno et al., 2006), procedures that should indeed be tested for osmoregulatory components of barnacle species representing a range of salinity tolerance. Similarly, more efforts should focus on immunohistochemical methods that employ fluorescence-labeled specific antibodies and high-resolution microscopy. However, the current main limitation is the production of species-specific antibodies to the rich plethora of osmoregulatory components.

In summary, we believe that by applying these new techniques and genomic resources, the underlying mechanisms in osmoregulation will be clarified by linking genes to physiology and evolution, which will be instrumental in providing better predictive models of barnacle ecology and evolution of adaptations to brackish environments.

## Author Contributions

All authors listed have made a substantial, direct and intellectual contribution to the work, and approved it for publication.

### Conflict of Interest Statement

The authors declare that the research was conducted in the absence of any commercial or financial relationships that could be construed as a potential conflict of interest.
