# Supplementary material for: Osmoregulation in Barnacles: An Evolutionary Perspective of Potential Mechanisms and Future Research Directions
Source: Front Physiol. 2019 Aug 21;10:877. doi: 10.3389/fphys.2019.00877 (PMC6712927; doi:10.3389/fphys.2019.00877)
Supplement: Supplementary file 1 [file Table_1.DOCX]

**Table S1**. Summary of experimental results for salinity tolerance in 20 barnacle species from 29 published reports. The table shows the geographic region for each study, the studied life-stage, the main response measured, the interval of tested salinities, the approximate range of salinity tolerance, and finally acclimation time. Note that the included studies used very different methods, life-stages and measured responses, and the salinity tolerance should be interpreted with caution.

| **Species** | **Region of study** | **Author** | **Life stage** | **Main physiological response** | **Salinity range (high)** | **Salinity range (low)** | **Salinity tolerated (high)** | **Salinity tolerated (low)** | **Acclimation time (h)** |
| --- | --- | --- | --- | --- | --- | --- | --- | --- | --- |
| Balanus amphitrite | USA, North Carolina | Crisp & Costlow (1963) | nauplius | hatching | 85 | 5 | 55.5 | 12.5 | 90 |
| Balanus amphitrite | India, Andhra Pradesh | Prasada Rao & Ganapati (1972) | adult | respiration | 32 | 5 | 32 | 10 | 0 |
| Balanus amphitrite | Japan, Sodeshi | Konya & Miki (1994) | cyprid | settlement | 65 | 0 | 35 | 14 | 0 |
| Balanus amphitrite | China, Hong Kong | Oiu & Qian (1999) | adults | survival | 35 | 10 | 35 | 10 | 0 |
| Balanus amphitrite | China, Hong Kong | Oiu & Qian (1999) | cyprid | survival | 35 | 10 | 35 | 10 | 0 |
| Balanus amphitrite | China, Hong Kong | Oiu & Qian (1999) | nauplius |  | 35 | 10 | 35 | 10 | 0 |
| Balanus amphitrite | Japan, Hamana Bay | Anil et al (1990) | nauplius | mortality | 30 | 10 | 30 | 10 | 0 |
| Balanus amphitrite | USA, San Fransisco | Newman (1967) | adult | cirral activity | 35 | 2.1 | 35 | 8.7 | 24 |
| Balanus balanus | UK, Isle of Man | Davenport (1976) | adult | cirral activity | 33 | 0 | 33 | 22.2 | 1 |
| Balanus balanus | UK | Barnes (1953) | nauplius | mortality | 33 | 2.1 | 33 | 22.5 | 48 |
| Balanus balanus | UK, Oban | Barnes & Barnes (1974) | nauplius | hatching | 60 | 0 | 40 | 25 | 48 |
| Balanus crenatus | UK, Menai Straits | Davenport (1976) | adult | cirral activity | 33 | 7.9 | 33 | 23.3 | 1 |
| Balanus crenatus | UK, Conway Castle | Davenport (1976) | adult | cirral activity | 33 | 7.9 | 33 | 19.2 | 1 |
| Balanus crenatus | UK, Conway Castle | Cawthorne (1979) | adult | closure | 33 | 0 | 33 | 18 | 48 |
| Balanus crenatus | UK, Menai Straits | Cawthorne (1979) | adult | closure | 33 | 0 | 33 | 20 | 48 |
| Balanus crenatus | UK | Barnes (1953) | nauplius | mortality | 33 | 3 | 33 | 15 | 48 |
| Balanus crenatus | UK, Oban | Barnes & Barnes (1974) | nauplius | hatching | 60 | 0 | 40 | 22.5 | 48 |
| Balanus crenatus | UK, Wales | Foster (1970) | adult | cirral activity | 55 | 0 | 55 | 13 | 168 |
| Balanus eburneus | USA, North Carolina | Crisp & Costlow (1963) | nauplius | hatching | 85 | 5 | 54.7 | 15.5 | 90 |
| Balanus eburneus | USA, Virginia | Dineen & Hines (1994a) | cyprid | settlement | 35 | 2 | 35 | 5 | 132 |
| Balanus glandula | USA, California | Bergen (1968) | embryo | mortality | 105 | 0 | 61.25 | 17.5 | 1 |
| Balanus glandula | USA, San Fransisco | Newman (1967) | adult | cirral activity | 35 | 2.1 | 35 | 17 | 24 |
| Balanus improvisus | UK, Tamar estuary | Davenport (1976) | adult | cirral activity | 33 | 7.8 | 33 | 7.9 | 1 |
| Balanus improvisus | UK, Conway Castle | Davenport (1976) | adult | cirral activity | 33 | 0 | 33 | 7.7 | 1 |
| Balanus improvisus | Sweden, Askö | Davenport (1976) | adult | cirral activity | 33 | 7.8 | 33 | 7.8 | 1 |
| Balanus improvisus | UK, Conway Castle | Cawthorne (1979) | adult | closure | 33 | 0 | 33 | 9.5 | 48 |
| Balanus improvisus | UK, Plymouth | Foster (1970) | adult | cirral activity | 55 | 0 | 55 | 2.5 | 144 |
| Balanus improvisus | USA, North Carolina | Fyhn (1976) | adult | survival, cirral activity | 40 | 0 | 35 | 0.24 | 1260 |
| Balanus improvisus | Iran, Caspian Sea | Nasrolahi et al (2006) | nauplius | survival | 36 | 7 | 24 | 7 | 0 |
| Balanus improvisus | Iran, Caspian Sea | Nasrolahi et al (2007) | cyprid | settlement | 36 | 7 | 36 | 7 | 0 |
| Balanus improvisus | Germany, Kiel | Nasrolahi et al (2012) | cyprid | survival | 30 | 5 | 30 | 5 | 0 |
| Balanus improvisus | Germany, Kiel | Nasrolahi et al (2012) | cyprid | settlement | 30 | 5 | 30 | 5 | 0 |
| Balanus improvisus | Germany, Kiel | Nasrolahi et al (2013) | juvenile | mortality | 30 | 5 | 30 | 5 | 0 |
| Balanus improvisus | USA, San Fransisco | Newman (1967) | adult | cirral activity | 35 | 1 | 35 | 2.1 | 24 |
| Balanus perforatus | UK, Oban | Barnes & Barnes (1974) | nauplius | hatching | 60 | 0 | 54 | 19.5 | 48 |
| Balanus reticulatus | India | Thiyagarajan et al. (2002) | cyprid | settlement | 40 | 20 | 40 | 20 | 24 |
| Balanus subalbidus | USA, Maryland | Dineen & Hines (1994b) | cyprid | metamorphosis | 35 | 2 | 30 | 2 | 144 |
| Balanus tintinnabulum | India, Andhra Pradesh | Prasada Rao & Ganapati (1972) | adult | respiration | 32 | 5 | 32 | 15 | 0 |
| Balanus trigonus | China, Hong Kong | Thiyagarajan et al. (2003) | nauplius | survival | 34 | 22 | 34 | 22 |  |
| Balanus trigonus | China, Hong Kong | Thiyagarajan et al. (2003) | cyprid | settlement | 34 | 22 | 34 | 26 | 144 |
| Chelonobia patula | USA, North Carolina | Crisp & Costlow (1963) | nauplius | hatching | 85 | 5 | 49 | 16 | 90 |
| Chirona hameri | UK, Isle of Man | Davenport (1976) | adult | cirral activity | 33 | 0 | 33 | 17.5 | 1 |
| Chirona hameri | UK, Isle of Man | Foster (1970) | adult | cirral activity | 35 | 17.5 | 35 | 25 | 1 |
| Chirona hameri | UK, Irish Sea | Cawthorne (1978) | nauplius | mortality | 36 | 8.75 | 36 | 11.0 | 24 |
| Chthamalus stellatus | UK, Bangor | Bhatnagar & Crisp (1965) | nauplius | mortality | 200 | 2 | 65 | 17 | 24 |
| Chthamalus stellatus | UK, Bangor | Bhatnagar & Crisp (1965) | nauplius | mobility | 200 | 2 | 45 | 17 | 24 |
| Chthamalus stellatus | UK, Oban | Barnes & Barnes (1974) | nauplius | hatching | 60 | 0 | 43 | 27 | 48 |
| Elminius modestus | UK | Cawthorne & Davenport (1980) | adult | closure | 33 | 0 | 33 | 21.7 | 6 |
| Elminius modestus | UK | Cawthorne & Davenport (1980) | cyprid | closure | 33 | 0 | 33 | 10 | 6 |
| Elminius modestus | UK, Menai Straits | Davenport (1976) | adult | cirral activity | 33 | 7.9 | 33 | 22.0 | 1 |
| Elminius modestus | UK, Conway Castle | Davenport (1976) | adult | cirral activity | 33 | 7.9 | 33 | 20.4 | 1 |
| Elminius modestus | UK, Conway Castle | Cawthorne (1979) | adult | closure | 33 | 0 | 33 | 21 | 48 |
| Elminius modestus | UK, Menai Straits | Cawthorne (1979) | adult | closure | 33 | 0 | 33 | 26 | 48 |
| Elminius modestus | UK, Aberffraw | Bhatnagar & Crisp (1965) | nauplius | mortality | 200 | 2 | 90 | 10 | 24 |
| Elminius modestus | UK, Aberffraw | Bhatnagar & Crisp (1965) | nauplius | mobility | 200 | 2 | 55 | 16 | 24 |
| Elminius modestus | UK, Oban | Barnes & Barnes (1974) | nauplius | hatching | 60 | 0 | 43 | 21.4 | 48 |
| Elminius modestus | UK, Wales | Foster (1970) | adult | cirral activity | 55 | 0 | 53 | 15 | 168 |
| Elminius modestus | UK, Southampton | Lance (1964) | nauplius | survival | 35 | 0 | 35 | 11 | 20 |
| Elminius modestus | UK, Menai Straits | Cawthorne (1978) | nauplius | mortality | 35 | 5.25 | 35 | 6.7 | 24 |
| Elminius modestus | Germany, Helgoland | Harms (1986) | nauplius | mortality | 50 | 10 | 50 | 20 | 0 |
| Elminius modestus | New Zeeland | Harms (1986) | nauplius | mortality | 40 | 20 | 40 | 20 | 0 |
| Fistulobalanus pallidus | Nigeria, Lagos | Sandison (1966) | adult | growth, survival | 30 | 0 | 16 | 1.5 | 720 |
| Semibalanus balanoides | UK | Cawthorne & Davenport (1980) | adult | closure | 33 | 0 | 33 | 21.7 | 6 |
| Semibalanus balanoides | UK | Cawthorne & Davenport (1980) | cyprid | closure | 33 | 0 | 33 | 8.7 | 6 |
| Semibalanus balanoides | UK, Menai Straits | Davenport (1976) | adult | cirral activity | 33 | 7.9 | 33 | 23.6 | 1 |
| Semibalanus balanoides | UK, Conway Castle | Cawthorne (1979) | adult | closure | 33 | 0 | 33 | 23 | 48 |
| Semibalanus balanoides | UK, Menai Straits | Cawthorne (1979) | adult | closure | 33 | 0 | 33 | 24 | 48 |
| Semibalanus balanoides | UK | Barnes (1953) | nauplius | mortality | 33 | 3 | 33 | 18 | 48 |
| Semibalanus balanoides | UK, Bangor | Bhatnagar & Crisp (1965) | nauplius | mortality | 200 | 2 | 80 | 15 | 24 |
| Semibalanus balanoides | UK, Bangor | Bhatnagar & Crisp (1965) | nauplius | mobility | 200 | 2 | 50 | 25 | 24 |
| Semibalanus balanoides | UK, Oban | Barnes & Barnes (1974) | nauplius | hatching | 60 | 0 | 40 | 18 | 48 |
| Semibalanus balanoides | UK, Wales | Foster (1970) | adult | cirral activity | 55 | 0 | 53 | 17 | 48 |
| Semibalanus balanoides | UK, Menai Straits | Cawthorne (1978) | nauplius | mortality | 35 | 8.75 | 35 | 8.2 | 24 |
| Tetraclita japonica | China, Hong Kong | Chan et al. (2001) | nauplius | mortality | 24 | 9 | 24 | 15 | 24 |
| Tetraclita squamosa | China, Hong Kong | Chan et al. (2001) | nauplius | mortality | 33 | 9 | 33 | 15 | 24 |
| Verruca stroemia | UK, Oban | Barnes & Klepal (1974) | adult | survival | 121 | 4.7 | 70 | 6.5 | 144 |
