# Supplementary material for: Osmoregulation in Barnacles: An Evolutionary Perspective of Potential Mechanisms and Future Research Directions
Source: Front Physiol. 2019 Aug 21;10:877. doi: 10.3389/fphys.2019.00877 (PMC6712927; doi:10.3389/fphys.2019.00877)
Supplement: Supplementary file 2 [file Table_2.DOCX]

**Table S2 - References cited in Table S1**

Anil, A. C., Chiba, K., & Okamoto, K. (1990). Macrofouling community structure and ecology of barnacles in Hamana Bay (Japan). *Biofouling, 2*, 137-150.

Barnes, H. (1953). The effect of lowered salinity on some barnacle nauplii. *Journal of Animal Ecology, 22*, 328-330.

Barnes, H., & Barnes, M. (1974). The response during development of the embryos of some common cirripedes to wide changes in salinity. *J Exp Mar Biol Ecol, 15*, 197-202.

Barnes, H., & Klepal, W. (1974). The general biology of *Verruca stroemi* (O.F. Müller). IV. Effect of salinity and temperature on survival, behaviour, and osmotic balance. *J Exp Mar Biol Ecol, 14*, 37-46.

Bergen, M. (1968). The salinity tolerance limits of the adults and early-stage embryos of *Balanus glandula* Darwin, 1854 (Cirripedia, Thoracica). *Crustaceana, 15*, 229-234.

Bhatnagar, K. M., & Crisp, D. J. (1965). The salinity tolerance of nauplius larvae of cirripedes. *Journal of Animal Ecology, 34*, 419-428.

Cawthorne, D. F. (1978). Tolerance of some cirripede nauplii to fluctuating salinities. *Marine Biology, 46*, 321-325.

Cawthorne, D. F. (1979). A comparative study of the closure response of some cirripede species exposed to falling seawater concentrations. *J. Mar. Biol. Ass. U.K., 59*, 811-817.

Cawthorne, D. F., & Davenport, J. (1980). The effect of fluctuating temperature, salinity, and aerial exposure upon larval release in *Balanus balanoides* and *Elminius* *modestus*. *J. Mar. Biol. Ass. U.K., 60*, 367-377.

Chan, B. K. K., Morritt, D., & Williams, G. A. (2001). The effect of salinity and recruitment on the distribution of *Tetraclita squamosa* and *Tetraclita japonica* (Cirripedia ; Balanomorpha) in Hong Hong. *Marine Biology, 138*, 999-1009.

Crisp, D. J., & Costlow, J. D. (1963). The tolerance of developing cirripede embryos to salinity and temperature. *Oikos, 14*, 22-34.

Davenport, J. (1976). A comparative study of the behaviour of some balanomorph barnacles exposed to fluctuating sea water concentrations. *J. Mar. Biol. Ass. U.K., 56*, 889-907.

Dineen Jr, J. F., & Hines, A. H. (1994a). Larval settlement of the polyhaline barnacle *Balanus eburneus* (Gould): cue interactions and comparisons with two estuarine congeners. *J Exp Mar Biol Ecol, 179*, 223-234.

Dineen Jr, J. F., & Hines, A. H. (1994b). Effects of salinity and adult extract on settlement of the oligohaline barnacle *Balanus subalbidus*. *Mar Biol, 119*, 423-430.

Foster, B. A. (1970). Responses and acclimation to salinity in the adults of some balanomorph barnacles. *Phil Trans R Soc Lond B, 256*, 377-400.

Fyhn, H. J. (1976). Holoeuryhalinity and its mechanisms in a cirriped crustacean, *Balanus improvisus*. *Comp Biochem Physiol, 53A*, 19-30.

Harms, J. (1986). Effects of temperature and salinity on larval development of *Elminius modestus* (Crustacea, Cirripedia) from Helgoland (North Sea) and New Zealand. *Helgoländer Meeresuntersuchungen, 40*, 355-376.

Kon-ya, K., & Miki, A. (1994). Effects of environmental factors on larval settlement of the barnacle *Balanus amphitrite* reared in the laboratory. *Fisheries Science, 60*, 563-565.

Lance, J. (1964). The salinity tolerance of some estuarine planktonic crustaceans. *Biol Bull, 127*, 108-118.

Nasrolahi, A., Farahani, F., & Saifabadi, S. J. (2006).

Effect of Salinity on Larval Development and Survival of the Caspian Sea Barnacle, *Balanus improvisus* Darwin (1854). *Journal of Biological Sciences, 6*, 1103-1107.

Nasrolahi, A., Pansch, C., Lenz, M., & Wahl, M. (2012). Being young in a changing world: how temperature and salinity changes interactively modify the performance of larval stages of the barnacle *Amphibalanus improvisus*. *Marine Biology, 159*, 331-340.

Nasrolahi, A., Pansch, C., Lenz, M., & Wahl, M. (2013). Temperature and salinity interactively impact early juvenile development: a bottleneck in barnacle ontogeny. *Marine Biology, 160*, 1109-1117.

Nasrolahi, A., Sari, A., Saifabadi, S., & Malek, M. (2007). Effects of algal diet on larval survival and growth of the barnacle *Amphibalanus* (=*Balanus*) *improvisus*. *Journal of the Marine Biological Association of the UK, 87*, 1227-1233.

Newman, W. A. (1967). *On physiology and behaviour of estuarine barnacles.* Paper presented at the Proceedings of Symposium on Crustacea Part 3: 1038-1066.

Prasada Rao, D. G. V., & Ganapati, F. N. A. (1972). Respiration in relation to salinity variation in intertidal barnacles. *Proceedings of the Indian National Science Academy, 38*, 425-429.

Qiu, J. W., & Qian, P. Y. (1999). Tolerance of the barnacle *Balanus amphitrite amphitrite* to salinity and temperature stress: effects of previous experience. *Marine Ecology Progress Series, 188*, 123-132.

Sandison, E. E. (1966). The effect of salinity fluctuations on the life cycle of *Balanus* *pallidus* *stutsburi* Darwin in Lagos Harbour, Nigeria. *J Anim Ecol, 35*, 363-378.

Thiyagarajan, V., Harder, T., & Qian, P. Y. (2003). Combined effects of temperature and salinity on larval development and attachment of the subtidal barnacle *Balanus* *trigonus* Darwin. *J Exp Mar Biol Ecol, 287*, 223-236.

Thiyagarajan, V., Nair, K. V. K., Subramoniam, T., & Venugopalan, V. P. (2002). Larval settlement behaviour of the barnacle *Balanus reticulatus* in the laboratory. *Journal of the Marine Biolgical Association of the UK, 82*, 579-582.
